# Implementation of LURIES: A New Handoff Tool for Pediatric Residents

**DOI:** 10.7759/cureus.2558

**Published:** 2018-04-30

**Authors:** Eva Seligman, Marcelo Malakooti

**Affiliations:** 1 Pediatric Emergency Medicine, Johns Hopkins Children's Center; 2 Division of Critical Care, Ann & Robert H Lurie Children's Hospital of Chicago/northwestern University Feinberg School of Medicine

**Keywords:** resident handoff

## Abstract

Introduction

At our institution, inpatient pediatric residents spend about 50 hours each month on 52 total handoff encounters. We created a modifiable electronic handoff tool (LURIES) and measured its impact on resident perception of self-efficacy. We also elicited feedback and identified topics omitted in the handoffs following its adoption on general medicine, intensive care, and sub-specialty wards. Our objective was to standardize the handoff tool used by residents and to measure the impact of the new tool on resident perception of self-efficacy.

Methods

Residents were trained to use LURIES and completed a voluntary online survey during the first and second year of implementation. Senior residents reflected on experiences prior to and after implementation of LURIES. Night team residents reported key items omitted from the handoff.

Results

65/96 (68%) residents responded in year one and 45/97 (46%) in year two of implementation. The majority of residents perceived that LURIES improved handoff efficiency, promoted active listening, and reduced the number of missed tasks overnight. There was a statistically significant increase in self-efficacy for all queried overnight tasks. Omitted information was most commonly identified on complex subspecialty services and pertained to communication with parents, late admissions, and discharges.

Conclusion

LURIES increased resident self-efficacy with patient management. There was no difference in findings when controlled for previous medical school handoff training or type of handoff tool used in medical school clerkships. More data are needed to establish the significance of this trend in relation to handoffs beyond residency, patient safety, and patient satisfaction scores.

## Introduction

In the era of resident work hour restrictions that necessitate frequent handoffs between providers, efficient and effective patient handoff methodology has become paramount in providing high-quality care [1]. At our institution, residents working on a typical inpatient service may spend over 50 hours on an average of 52 handoff encounters per month (given 26 shifts per month times 60 minutes per handoff session, per internal programmatic data). As residents from the day and night teams relay key patient information to one another, continuity of patient care may be threatened by disrupted communication cascades [2]. Additionally, a resident may be asked to attend to an admission, return to the bedside of a critically ill patient, or answer a time-sensitive nursing question during the period designated for handoffs [3]. With these competing interests for time and attention [3, 4], a streamlined and comprehensive handoff is required for optimal communication between physicians.

**Figure 1:**
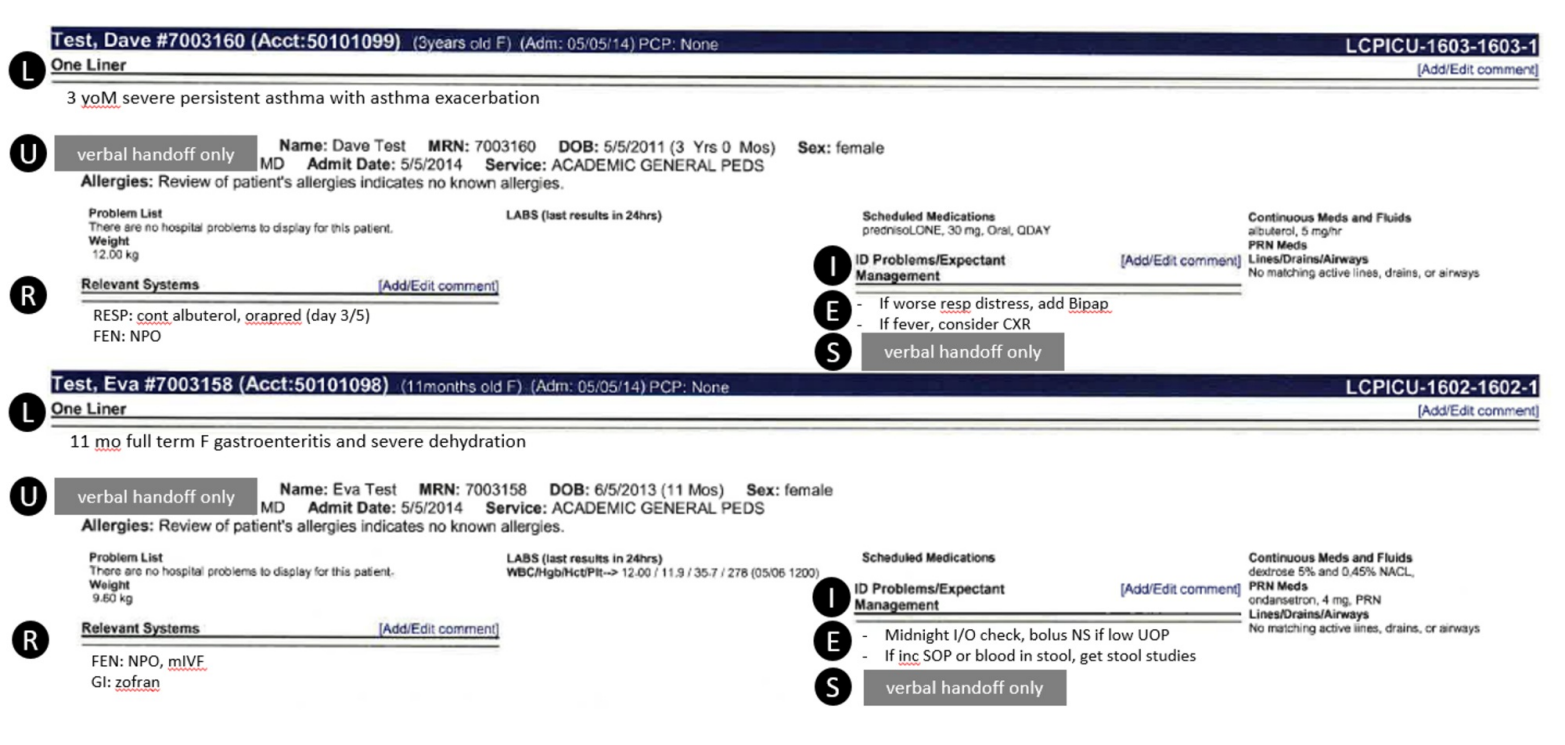
Example LURIES EMR tool. Letters of the mnemonic are labeled in circles for demonstration purposes only and do not appear in the EMR. Content of the mnemonic "L," "R," and "I/E" is typed directly into the EMR and stated verbally during handoff. Content "U" and "S" are part of the verbal handoff only. EMR: Electronic medical record

In general, the use of standardized handoff mnemonics improves perceptions of uncertainty about decisions made overnight, accuracy of handoff information, and reduces medical errors [5,6]. To date, few studies have investigated the impact of computerized handoff tools on residents' perceptions of self-efficacy. At Ann & Robert H. Lurie Children's Hospital of Chicago, we created a modifiable electronic tool for residents that follows the verbal mnemonic, LURIES (Table 1), which is directly incorporated into the electronic medical record (EMR) shown (Figure 1). We hypothesized that resident self-efficacy and confidence in completing common overnight tasks would improve with the residency-wide adoption of this tool on general medicine, intensive care, and sub-specialty wards at our urban, academic, pediatric tertiary care center.

**Table 1:** LURIES mnemonic.

LURIES	
L	One-Liner with statement of severity
U	Updates from the day
R	Relevant systems
I	Identify overnight issues/tasks
E	Expectant management
S	Summary/Synthesis

## Materials and methods

To test this hypothesis we implemented a novel handoff tool with easy recall that was streamlined within the existing EMR. After adoption of LURIES among inpatient wards teams, we identified via anonymous survey resident satisfaction with the tool, its impact on resident perceptions of self-efficacy and patient safety. We also sought to identify key items missed in the handoff and to elicit resident feedback on education and functionality of the tool.

This study was conducted over two academic years. Prior to initiation of this study, all pediatric residents received training on the use of a new, electronic modifiable handoff tool in the EMR (Epic, Verona, WI, USA) and on the verbal mnemonic, LURIES, as part of orientation to the new academic year. Day residents were taught to verbalize the components of the mnemonic to incoming night residents and to complete the corresponding written tool in the EMR. Information such as vital signs, medications, and recent laboratory values populated automatically into the handoff tool, while residents were instructed to manually update the patient summary, relevant systems, and expectant management fields on a daily basis. The educational program included a didactic session, best practice video demonstration, use of the handoff mnemonic, and case-based simulations of the common pediatric admissions ("asthma exacerbation" and "fever in a neonate"). The principal investigator, coinvestigators, and chief residents were present during the simulations to observe and provide immediate verbal feedback to residents regarding their use of the mnemonic.

Pediatric residents were annually consented to participate in a voluntary survey. The surveys were available electronically and in paper format, and included Likert-scale, true-false, and open-ended questions. The first survey occurred three months after initial implementation of LURIES. In this survey, post-graduate year (PGY)-II and PGY-III residents were asked to complete two sets of questions: one to reflect on their experience during prior years (before implementation of LURIES), and one to reflect on their current experience. At the start of the next academic year, the investigators administered an expanded LURIES training session incorporating data from the initial survey and including both practice cases and immediate in-person feedback. Three months into the second year of use of LURIES, residents were again asked to complete a similar survey. In addition to this second survey, residents on the night team also completed an anonymous form indicating when an event occurred overnight perceived as not appropriately accounted for based on information within handoff tool. These responses were analyzed by specialty service and topic of missed item. Wilcoxon Signed-Rank test was used to examine differences in responses prior to and after the introduction of modifiable handoff tool. Wilcoxon Rank-Sum and/or Kruskal-Wallis tests examined differences in median individual responses by level. There were no type I error adjustments made for multiple comparisons, and all analyses assumed type I error rate of 5%. For dichotomous (true/false) items, scores were summarized via sample proportions and respective 95% confidence limits.

## Results

**Figure 2:**
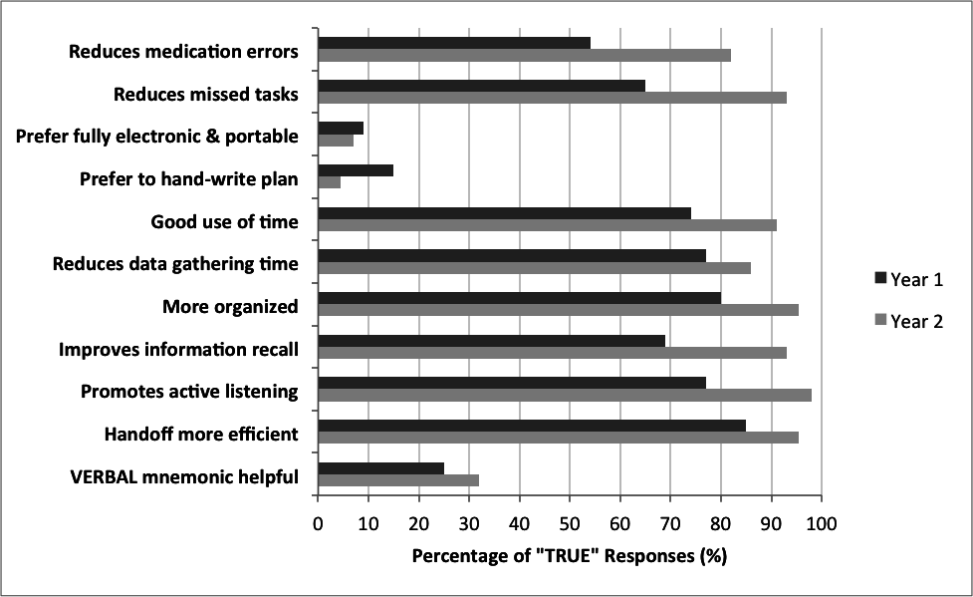
Affirmative survey responses to true/false questions, all PGY years. Abbreviated stems of relevant survey questions, demonstrating the percentage of “TRUE” responses combined across all queried residents in both Year 1 and Year 2 surveys. There is an increase in the percentage of residents with affirmative responses for all questions from Year 1 to Year 2 in favor of the LURIES method. The two questions with lower “TRUE” response rates in Year 2 include preferences for hand written plans and for fully electronic methods, i.e., both in favor of the LURIES predominantly electronic integrated tool, with ability to print hard copies. PGY: Post-graduate year

**Figure 3:**
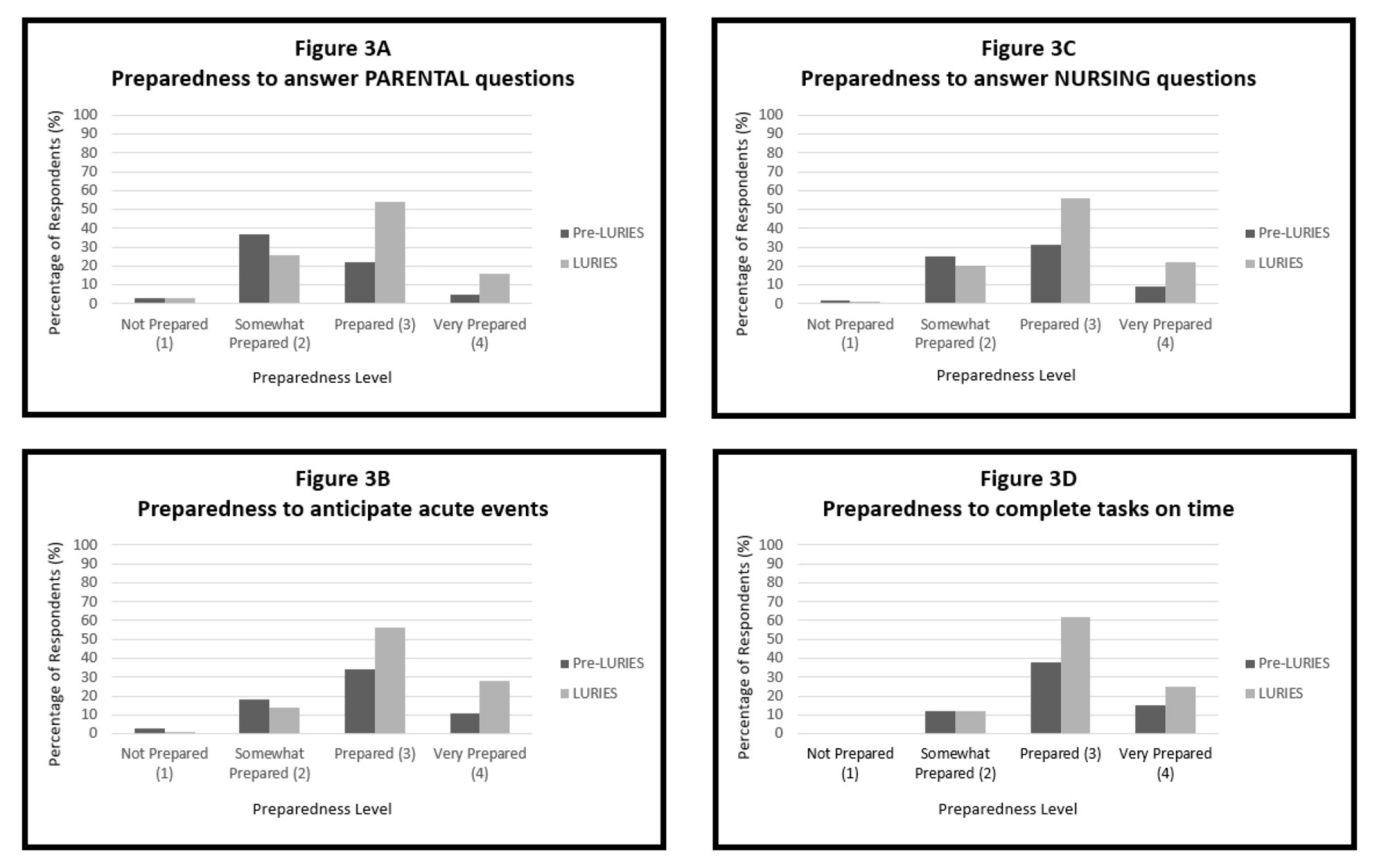
Resident self-efficacy by survey response: across four domains of patient care overnight (A-D), for all PGY levels. “Pre-LURIES” refers to efficacy scores prior to implementation of the handoff tool. "LURIES" refers to aggregate responses from the first and second year surveys following handoff the tool implementation. Comparing pre-LURIES and LURIES responses, the median score for all four domains improved (i.e., higher self-efficacy). PGY: Post-graduate year

In year one, 65/96 (68%) of all current pediatric residents responded to the survey. Respondents were 31% PGY-I, 34% PGY-II, and 35% PGY-III level (Table 2). In the first year after implementation, 87% of respondents indicated that LURIES improved efficiency of the handoff process compared to prior handoff technique (Figure 2), 77% stated that it promoted active listening, 65% had increased confidence in ability to complete tasks overnight, and 65% perceived that LURIES reduced the number of missed tasks overnight (Figure 2). During the first year of implementation, first and second year resident responses were more likely to be supportive of the electronic handoff tool when compared to the responses of third-year residents. The percentage of residents who felt prepared (response score of 3) or very prepared (response score of 4) increased following implementation of LURIES. For all questions, the median response score improved (i.e., higher self-efficacy): preparedness to “complete tasks on time,” median score 3.0 vs. 3.2, p < 0.01 (Figure 3D); “answer parent questions about the plan,” median score 2.4 vs. 3.1, p < 0.01 (Figure 3A); “answer nursing questions about the plan,” median score 2.7 vs. 3.1, p < 0.01 (Figure 3C); and, “anticipate acute events that could occur overnight,” median score 2.8 vs. 3.3, p < 0.05 (Figure 3B). In subgroup analysis comparing year one to year two responses, there was no statistical increase in median score.

**Table 2:** Demographics of respondents. The distribution of respondents by level of training. N is the number of individuals. PGY: Post-graduate year

	Year 1 N (%)	Year 2 N (%)
PGY-I	20 (31)	20 (45)
PGY-II	22 (34)	11 (24)
PGY-III	23 (35)	14 (31)

In year two, 45/97 (46%) of pediatric residents responded to the survey, most were members of the PGY-I class (Table 2). Decreased response rates in the PGY-II and III classes likely account for the decreased overall response rate in year two (these residents had participated in the initial survey and chose not to participate in the survey the second year). Overall satisfaction with the tool improved in the survey obtained during the second year of implementation, and perception of preparedness did not vary significantly when compared to the year one survey responses (Figure 2). While the majority of residents had experience using an electronic handoff tool in medical school, most had not received formal handoff training (Table 3). There was no difference in findings after controlling for prior medical school handoff training or type of handoff tool used in medical school clerkships. In the open-ended "comments" area of the survey, many residents (18/97, 19%) identified a need for more formalized handoff training, including the opportunity to observe seasoned clinicians perform handoffs and a need for more direct feedback.

**Table 3:** Handoff experience in medical school. The raw number (N) and proportion of residents who were exposed to handoff training and electronic tools during medical school (prior to residency).

	Received handoff training in medical school	Used electronic handoff tool in medical school
Intern Year	N (%)	N (%)
2012-2013	6 (27)	12 (54)
2013-2014	9 (40)	16 (73)
2014-2015	7 (39)	9 (50)
2015-2016	9 (47)	8 (42)

The resident-reported omissions survey was open for six months during year two. Although teams had reduced resident staffing on the weekends, only 19% of omissions occurred during that period. Most of the omissions were regarding communication with parents, social concerns, or issues surrounding late admissions or discharges (Table 4). The response rate was too small to power statistical analysis.

**Table 4:** Self-reported incidence of omitted tasks overnight during use of LURIES. Overnight residents were polled via anonymous electronic survey to “report issues that arise overnight that were not included in the handoff.” The responses are displayed by content theme of the omission.

Common themes of omissions	Number of events
Parent communication/social	3
Fever	2
Hypertension	2
Diet	2
Lines/Procedures	2
Admission/Discharge	3
Lab schedule	2

## Discussion

We demonstrated that the introduction of a standardized, electronic handoff tool with a corresponding verbal mnemonic had a strong impact on residents’ ability to care for patients, particularly via improving self-efficacy. For example, there was a 44% increase in resident perception of being "prepared" or "very prepared" to anticipate acute events overnight, and a 41% increase in being "prepared" or "very prepared" to answer nursing questions.

The concept of self-efficacy was first described in the 1970s as an individual’s belief in her capability to organize and execute a plan to manage future situations [7]. Self-efficacy predicts academic performance in medical students [7] and is correlated with resident performance in simulated patient care scenarios [8]. While self-efficacy levels are not without limitations in correlating to performance [9, 10], use of LURIES increased the resident perception of self-efficacy and comfort with patient management while caring for patients overnight, discussing management plans with parents and nursing staff, and anticipating acute events, leading to widespread use of the tool.

After controlling for prior medical school handoff training or type of handoff tool used in medical school clerkships, these results were preserved, highlighting the importance of consistent handoff training during residency, regardless of medical school education. In our experience, residents readily began completing the EMR-modifiable sections of LURIES and incorporated this task into their daily work flow. In contrast, changing the language used during the corresponding verbal handoff was not consistently adopted, which may have been a marker of slow culture shift in the setting of a new, more structured format. Since the conclusion of this study, however, LURIES was established as the mandatory handoff procedure for all incoming and current residents. It is used consistently among all pediatric residents at our institution. The sustained successful implementation of LURIES requires an infrastructure for feedback and continuing education [5,11,12]. We provided ongoing LURIES education and orientation for new trainees, and are in the process of developing a mechanism to provide formal observation and feedback to residents throughout the year. Results of handoff observations and feedback will provide additional insight into ways that the annual formal handoff curriculum should be revised.

Despite emphasis on standardized handoffs among residents, adoption of a standardized handoff method has not yet been formally incorporated by other providers at our center, such as attending physicians and non-physician care providers. Anecdotally, we have observed that physicians often discontinue use of formalized handoff procedures as they advance in practice, and use of any such tool among mid-level providers is varied. The profound reduction of medical errors demonstrated in previous studies, in addition to the immediate improvement in self-efficacy demonstrated here, would suggest the potential for standardized handoffs to improve patient care and communication beyond residency [6]. We anticipate widespread adoption in the near future as institutional emphasis on standardized handoffs increases. Future studies will investigate the perception of handoffs by more advanced providers as well as how the use of a standardized electronic handoff during residency translates into practice after graduation.

This study was primarily limited by recall bias due to the self-reporting survey format. Self-perception data also may not accurately reflect actual experience. Additionally, this study had a small sample size, was limited to a single institution, and had a short follow up time frame. Furthermore, statistical analysis for data collected on tasks omitted from the handoff was underpowered due to low response rate.

## Conclusions

Introduction of a standardized electronic handoff tool incorporated directly into the existing EMR and with a corresponding verbal mnemonic readily improved resident self-efficacy when working on the wards overnight. More data are needed to establish the significance of this trend in relation to patient safety events and patient satisfaction scores. There is a need for yearly education about handoffs with a focus on portions of the care plan that are omitted most frequently as well as the use of the verbal mnemonic. Future studies will examine the use and impact of standardized handoffs beyond residency.

## References

[1] DeRienzo CM, Frush K, Barfield ME (2012). Handoffs in the era of duty hours reform: a focused review and strategy to address changes in the Accreditation Council for Graduate Medical Education Common Program Requirements. Acad Med.

[2] Woolf SH, Kuzel AJ, Dovey SM, Phillips RL Jr. (2004). A string of mistakes: the importance of cascade analysis in describing, counting, and preventing medical errors. Ann Fam Med.

[3] Kowitlawakul Y, Leong B, Lua A (2015). Observation of handover process in an intensive care unit (ICU): barriers and quality improvement strategy. Int J Qual Health Care.

[4] Rivera-Rodriguez JA, Karsh B-T (2010). Interruptions and distractions in healthcare: review and reappraisal. BMJ Qual Safety.

[5] Starmer AJ, O'Toole JK, Rosenbluth G (2014). Development, implementation, and dissemination of the I-PASS handoff curriculum: a multisite educational intervention to improve patient handoffs. Acad Med.

[6] Starmer AJ, Sectish TC, Simon DW (2013). Rates of medical errors and preventable adverse events among hospitalized children following implementation of a resident handoff bundle. JAMA.

[7] Dybowski C, Kriston L, Harendza S (2016). Psychometric properties of the newly developed Physician Teaching Self-Efficacy Questionnaire (PTSQ). BMC Med Educ.

[8] Malakooti MR, McBride ME, Mobley B, Goldstein JL, Adler MD, McGaghie WC (2015). Mastery of status epilepticus management via simulation-based learning for pediatrics residents. J Grad Med Educ.

[9] Riesenberg LA, Leitzsch J, Massucci JL (2009). Residents' and attending physicians' handoffs: a systematic review of the literature. Acad Med.

[10] Nadel FM, Lavelle JM, Fein JA, Giardino AP, Decker JM, Durbin DR (2000). Assessing pediatric senior residents’ training in resuscitation: fund of knowledge, technical skills, and perception of confidence. Pediatric Emergency Care.

[11] Gordon M, Findley R (2011). Educational interventions to improve handover in health care: a systematic review. Med Educ.

[12] Wayne DB, Butter J, Siddall VJ, Fudala MJ, Wade LD, Feinglass J, Mcgaghie WC (2006). Graduating internal medicine residents’ self-assessment and performance of advanced cardiac life support skills. Med Teach.

